# The Coronavirus (COVID-19) Pandemic and Mental Health of African Immigrants in the United States

**DOI:** 10.3390/ijerph191610095

**Published:** 2022-08-15

**Authors:** Korede Kafayat Yusuf, Ednah Madu, Salome Kutchava, Sun Kei Liu

**Affiliations:** College of Nursing and Public Health, Adelphi University, One South Avenue, Garden City, NY 11530, USA

**Keywords:** COVID-19, mental health, African immigrants, depression, anxiety

## Abstract

The impact of the COVID-19 pandemic on immigrants in the United States is understudied. We investigated the effect of the pandemic on the mental health of African immigrants in the United States and if subjective religiosity was a protective factor. We analyzed primary data collected using an online survey (*n* = 260). The study’s outcome variable was incident mental health disorder associated with the pandemic, and the primary independent variable was religiosity. Chi-squared and Mann–Whitney U tests were used to compare the distribution of variables by incident mental health disorders. Logistic regression models were used to quantify the association between predictors and the outcome. There were 39 (15.0%) new cases of mental health disorders related to the pandemic. The median scores in the three domains of religiosity of those who developed a mental health disorder and those who did not were not significantly different. Significant predictors of this outcome included having a strong religious support network and loss of household employment income. African immigrants experienced an increased rate of mental health disorders during the pandemic. Implementing community-based strategies to provide social support during a disaster may be essential in promoting mental health in the African immigrant community.

## 1. Introduction

The novel coronavirus disease 2019 (COVID-19) was declared a pandemic in 2020. It has affected the health of all populations, with almost five hundred million infections and over six million deaths to date [1]. The world’s first case was reported in Wuhan, China, in December 2019 and quickly spread all over the world throughout the winter months. As the incidence of cases increased, the world went into lockdown. Consequently, there was mandated social isolation, travel restriction, the closing of borders, a stoppage of the economy, and skyrocketing unemployment rates. The pandemic has caused not only long-term socioeconomic consequences but also physical and psychological health impacts [2]. Since declared a pandemic, COVID-19 has led to an increase in the incidence of mental health illnesses, including depression, post-traumatic stress disorder (PTSD), substance abuse disorder, domestic violence, and child abuse in the general population [2].

The impact of the pandemic on immigrants in the United States is understudied. Immigrants, a vulnerable population, are generally at increased risk for poor physical, psychological, and social health outcomes [3]. Immigrants face overwhelming stress in their acculturation process to the new society [4], and this has been linked to the manifestation of psychiatric disorders [5]. Immigrants are not favored in many essential aspects of health, such as insurance coverage, health care utilization, health care quality, employment opportunities, language, and health awareness [6]. Furthermore, the fear of the deadly COVID-19 virus and the consequences of the pandemic, including social isolation, job losses, and economic uncertainty, create an additional burden, which can worsen the health outcomes in this vulnerable population. Of concern are Black immigrants, who are already at a disadvantage due to the high prevalence of chronic medical conditions [7]. This racial minority group represents a rapidly growing subset of the US immigrant population. Between 2010 and 2018, the sub-Saharan African immigrant population increased by 52%, significantly outnumbering the 12% growth rate for the overall foreign-born population during that same period [8].

Religion is essential to immigrants as it offers a social context to connect with other people from similar ethnic backgrounds [9]. Religious services provide a place to worship in the immigrants’ native language and are a source of psychological and instrumental support for recent immigrants who need health care, jobs, and housing [10]. Several studies have shown that religiosity, spirituality, or regular religious participation is associated with better emotional or mental health outcomes [11,12]. Religious involvement better aids immigrant emotional well-being compared to non-religious groups with equal levels of social interaction [7]. As a result, the role of religiosity in preventing adverse mental health outcomes is worth investigating, particularly during a widespread disaster such as the COVID-19 pandemic.

In this study, we investigated the effect of the pandemic on the mental health of African immigrants in the United States, and if subjective religiosity was a protective factor. We are not aware of any US study that has examined the pandemic’s impact on this population.

## 2. Materials and Methods

In this cross-sectional study, we analyzed primary data (*n* = 260) that were collected to assess the effect of COVID-19 on the psychological and physical health of African immigrants in the United States. A quantitative survey was administered through the Qualtrics online survey platform from 15 April to 28 July 2021. The survey was pre-tested using a small convenience sample from the target population. Eligible participants were Sub-Saharan African immigrant adults aged ≥18 years, able to read English and residents of the United States. Participants were recruited through established networks of immigrant and faith-based organizations and snowball sampling. The community and religious leaders were identified through an online search. Following this, they were contacted and provided with information about the study via email. Their support was solicited in disseminating the survey and encouraging the community members to participate in the study. The leaders were informed to distribute the online survey via email and text message to their congregation or community members. Online surveys are one of the most widely utilized survey methods and the most feasible considering the ongoing pandemic and the resultant social distancing measures.

The potential participants received the recruitment script with information on the study’s purpose and eligibility requirements. They were screened for eligibility, and those who met the inclusion criteria were able to take the survey. Informed consent was obtained via Qualtrics before survey completion, and participants were entered into a raffle to win one of ten USD 20 electronic gift card incentives after completing the survey. The questionnaire was designed using validated tools to measure mental and physical health, religiosity, the experience of discrimination, social support, sleep, acculturation, sociodemographic characteristics, and other significant covariates. COVID-19 knowledge, attitude, and religious practice-related questions were also asked. The study was approved by the institutional review board of Adelphi University.

### 2.1. Variables

The study’s outcome variable was incident mental health disorder, defined as a self-report of depression, anxiety or anxiety disorder, suicidal thoughts, or any other mental health condition associated with the pandemic. To identify incident mental health disorders, we compared participants’ responses to the questions “Before the onset of the coronavirus pandemic, did you suffer any of the following health problems: (a) depression, (b) anxiety or anxiety disorder (including acute stress disorder, anxiety, generalized anxiety disorder, obsessive-compulsive disorder, panic disorder, phobia), (c) suicidal thoughts, or (d) any other mental health condition?” and “Since the coronavirus pandemic started, have you suffered any of the following health problems (list above was repeated)?” Individuals who reported experiencing any of the identified mental health disorders only after the start of the pandemic were classified as a case of incident mental health disorder.

The independent variable, religiosity, was assessed using the Duke University Religion Index (DUREL). The DUREL was designed to measure religiosity in Western religions such as Christianity and Islam [13]. The five-item scale assesses the three major dimensions of religious involvement: organizational, non-organizational, and intrinsic or subjective religiosity. Organizational religious activity (ORA) involves public religious activities such as attending religious services or participating in other group-related religious activities such as scripture study groups. Non-organizational religious activity (NORA) consists of religious activities performed in private, such as prayer and watching religious TV programs. Intrinsic religiosity (IR) assesses the degree of personal religious commitment or motivation [13]. Religiosity was evaluated based on the three separate subscales examined independently, with higher scores indicating greater ORA, NORA, and IR. DUREL has been widely used in research, and the overall scale has high test–retest reliability (intra-class correlation = 0.91) and high internal consistency—Cronbach’s alpha of 0.78–0.91 [13].

The covariates examined in this study include sociodemographic factors (age, gender, highest educational level, employment status, household income, marital status, insurance), length of stay in the US, physical activity, sleep, body mass index, cigarette smoking, religious social support, the experience of discrimination, any chronic medical condition, and COVID-19 related questions (infection, death, a loss of employment income in the household).

### 2.2. Statistical Analysis

Study variables were described using counts and proportions with 95% confidence intervals for categorical variables, and median and interquartile ranges for continuous data. All variables were checked for missing data, and the sociodemographic characteristics of participants with missing and complete data on the main exposure and the outcome variables were compared. Data analysis was restricted to individuals who had no missing data on the exposure and the outcome variables (*n* = 260). The incident cases of mental health disorders associated with the pandemic and the point prevalence of these disorders were computed. A chi-squared (χ2) test was used to compare the distribution of categorical variables by incident mental health disorders, while continuous variables were compared using the Mann–Whitney U Test. Bivariate and multivariate logistic regression analyses were used to quantify the association between religiosity and incident mental health disorders. Statistically significant predictors identified from the bivariate analyses were introduced into the multivariate regression models as confounders. Three models were conducted, one for each of the dimensions of religious involvement. For the covariates, measures of association were similar across the three models, and results were reported from the first model with organizational religious activity (ORA) as the primary exposure variable. The results are indicated as unadjusted odds ratios (ORs) and adjusted odds ratios (AORs) with corresponding 95% confidence intervals (CI). All hypothesis tests were two-tailed, and results were considered statistically significant with a type-1 error rate set at 5%. SPSS version 28 was used for statistical analysis.

## 3. Results

A total of 297 individuals responded to the survey, and we analyzed data for 260 (87.5%) participants with complete data. There were no statistically significant differences in the sociodemographic characteristics of participants with missing data and those with complete data on the study’s exposure and the outcome variables (data not presented). The analytic sample included participants recruited from 24 US states. 

Of these African immigrants, 156 (60.0%) had a graduate degree, 104 (40.0%) had an annual household income of at least USD 100,000, and 72 (27.7%) experienced a loss of employment income because of the coronavirus pandemic (Table 1). Approximately half of the participants (49.7%) have lived in the US for greater than 10 years. Their religious affiliation was mostly Protestant or Pentecostal (39.6%) and Islam (34.6%). Participants’ DUREL scores showed the full range of possible scores for each of the three subscales. Most participants had a high score of at least five for organizational (73.0%) and non-organizational (61.1%) religiosity. For intrinsic religiosity, almost half (47.1%) of the study sample had the highest possible of 15.

The overall prevalence of any mental health disorder in this population was 28.8 percent: depression (13.1%), anxiety/anxiety disorder (17.3%), suicidal thoughts (2.7%), and any other mental health condition (2.7%). Thirty-six participants (13.8%) reported a mental health disorder before the pandemic, and there were thirty-nine (15.0%) new cases of a mental health disorder related to the pandemic. This indicates a 109% increase in the rate of self-reported mental health disorders during the pandemic. Of the 39 new cases of a mental health disorder, 53.8% were self-reported depression, anxiety/anxiety disorder (64.1%), suicidal thoughts (12.8%), and any other mental health condition (7.7%).

Table 2 shows the results of the bivariate analysis of the determinants of incident mental health disorders. The median scores in the three domains of religiosity (organizational, non-organizational, and intrinsic) were the same between those who developed a mental health disorder and those who did not. The median score (IQR) for organizational, non-organizational, and intrinsic religiosity were 5.0 (2.0), 5.0 (1.0), and 14.0 (3.0), respectively. Individuals who developed a mental health disorder were significantly younger than those who did not (mean age of 37.9 vs. 42.7 years). Participants who reported pandemic-related mental health disorders were more likely to be unmarried (*p*-value—0.021), consume alcohol (*p*-value—0.036), and not have a strong religious support network (*p*-value—0.020). Other significant predictors of this outcome included loss of employment income in the household because of the coronavirus pandemic (*p*-value—0.005) and current unemployment status (*p*-value—0.027). Although individuals who had lived in the US for less than 10 years were more likely to report incident mental health disorders, length of stay in the US was not significantly associated with mental health (*p*-value—0.300).

In Table 3, we present the results of the unadjusted and adjusted logistic regression analysis of the significant predictors of incident mental health disorders. Religiosity was not significantly associated with pandemic-related mental health in both the unadjusted and adjusted models. In the unadjusted models, compared to individuals with a strong religious support network, those without a strong religious support network had 2.4 times higher odds of incident mental health disorders (unadjusted odds ratio: OR = 2.41, 95% CI = 1.13–5.11). Participants who consumed alcohol had two times higher odds of incident mental health disorders than non-consumers of alcohol (OR = 2.11, 95% CI = 1.04–4.29). Compared to married individuals, having never been married (OR = 2.30, 95% CI = 1.04–5.08) or of “other” marital status increases the odds of incident mental health disorders (OR = 3.13, 95% CI = 1.16–8.42). Being unemployed (OR = 2.25, 95% CI = 1.08–4.69) or having someone in the household with a loss of employment income because of the coronavirus pandemic (OR = 2.65, 95% CI = 1.32–5.34) were also significantly associated with higher odds of reporting incident mental health disorders. In the adjusted model, only a strong religious support network and having someone in the household with a loss of employment income because of the coronavirus pandemic remained significant predictors of incident mental health disorders. African immigrants without a strong religious support network had over 2.6 times higher odds of incident mental health disorders than those with a strong religious support network (adjusted odds ratio: AOR = 2.65, 95% CI = 1.02–6.92). Compared to individuals without anyone in the household with a loss of employment income because of the coronavirus pandemic, those who had someone in the household with a loss of employment had almost 2.6 times higher odds of incident mental health disorders (AOR = 2.59, 95% CI = 1.21–5.53).

## 4. Discussion

In the current study, we found that a quarter (25%) of the participants reported a mental health disorder during the pandemic, including 15% of new cases of a mental health disorder related to the pandemic. We defined the study outcome, incident mental health disorder, as a self-report of depression, anxiety or anxiety disorder, suicidal thoughts, or any other mental health condition associated with the pandemic. Based on the DUREL scores, our study participants predominantly have a high level of religiosity. Religiosity was not associated with pandemic-related mental health disorders but having a strong religious support network was. The other significant predictor of this outcome was a loss of household employment income because of the pandemic. Our study aligns with recommended additional research, which pays systematic attention to stress proliferation processes such as stressful experiences linked to natural or man-made environmental crises, particularly among immigrants and people of color, as well as cataloging and quantifying protective factors and enhancing knowledge on the associations between physical and mental health [14]. In consideration of the unique racial, immigration, and socio-cultural challenges confronting African immigrants, understanding the influence of COVID-19 and the role of religiosity is a worthy cause. 

The literature documents the enormous impact of the pandemic on overall health, including mental health. Higher than normal levels of depression, and anxiety, have been reported since the outbreak of COVID-19 [15,16], and the degree of increase differs among different subpopulations [15,17]. However, we did not find any studies that examined mental health in African immigrant subpopulations. In this cross-sectional study, we observed an increase in the incidence of mental health disorders among African immigrants during the pandemic. The rate of self-reported mental health disorders, including depression, doubled in this population. Similar to our findings, a recent study found that the prevalence of depressive symptoms in the general United States population more than tripled during the pandemic. Though the rate of increase is lower in our study, it is significant and worthy of further investigation. Mental health problems such as anxiety and depression in the African immigrant population may be caused by worry about becoming infected [18], economic uncertainty, prolonged social isolation [19], and co-existing immigration-related acculturation issues [4]. 

Similar to the African immigrant group in the current study, the prevalence of mental health increased in the African American communities during the COVID-19 pandemic and was linked to systemic racism and psychosocial consequences of the COVID-19 pandemic itself [20,21]. The high prevalence of mental health issues during the pandemic was also noted in Hispanic communities [22]. It is important to note that tools different from ours were used to measure mental health in the cited studies, thereby limiting the direct comparison of findings. In a mixed-method study, Garcini et al. [22] identified six themes for substantial distress and mental health concerns among underserved Latinx communities during the COVID-19 pandemic. These themes included economics, immigration, social isolation, misinformation, family, and health. One could argue that some of these factors, particularly socioeconomic challenges, and social isolation, may account for increased mental health issues during the COVID-19 pandemic. Qualitative studies to gain an in-depth understanding of the reasons for a higher rate of mental health disorders among African immigrants, including the role of racism and discrimination, are required.

Religion has played a crucial role in the resilience of many racial and ethnic minority populations. A religion’s promotion of perseverance and faith has aided individuals in times of hardships and inequality [23]. A study conducted prior to the pandemic found a significant relationship between religiosity and impulsiveness [24]. Their findings suggest that mental illness might impair religious involvement, and religiosity could reduce how people express mental illness or display impulsive behaviors. Our current study found no association between religiosity and mental health. Similar to our findings, Ouanes et al. [25] did not observe significant associations between religiosity and mental health in a Qatar population during the pandemic. Contrary to our results, a positive association between religiosity and mental health was found through divergent means in another study conducted in the pandemic era. Kranz et al. [26] found a positive relationship between religiosity and a problematic affective reaction to the coronavirus crisis (somatic anxiety). A possible reason for the lack of association observed in the current study might signify that religiosity by itself is not sufficient to promote mental health in this population during a disaster. 

Although religiosity was not significantly associated with mental health in our study, having a strong religious support network was. Finding religious support as a protective factor of mental health is consistent with the current literature. In a study conducted before the pandemic, Holt et al. [27] found that positive religious support was associated with lower depressive symptoms. Additionally, in a review of the literature, Serafini et al. [16] reported that a higher perception of social support was associated with a lower likelihood of developing psychological distress and psychiatric conditions. These findings have implications for mental health interventions in faith-based organizations within African immigrant communities. The provision of social support rather than promoting spirituality or religiosity during a social crisis such as a disease outbreak may help to promote mental health.

The other significant predictor of mental health issues in this study was a loss of employment income in the household because of the coronavirus pandemic. Using a Kenyan sample, Pinchoff et al. [28] also found a positive relationship between adult loss of income and depressive symptoms during the COVID-19 pandemic. The US Census Bureau’s Household Pulse Survey also reported that adults in households with job loss or lower incomes during the pandemic had higher rates of symptoms of mental illness than those without job or income loss. [29]. Additionally, households that experienced income or job loss were significantly more likely to report that worry or stress over the coronavirus pandemic had adversely impacted their mental health [29]. Loss of employment income in the household, particularly during times of economic crisis, can create emotional distress and consequent mental health issues. This finding highlights the importance of considering the household loss of employment income as a key factor when planning strategies to improve mental health during and after the COVID-19 pandemic. 

One of the limitations of this study is the small sample size. Factors such as marital status and alcohol use that became insignificant in the multivariate analysis may truly be associated with mental health in this population. The lack of significant association might be due to low power. A larger study will be needed to better understand the impact of the pandemic on the African immigrant community. We utilized a cross-sectional study design which limits the assessment of the impact of the pandemic on the outcome. A longitudinal study will be required to study the long-term effect of the pandemic on mental health and establish causal relationships. Our study participants were recruited mainly via faith-based and community organizations. The use of a convenient sample could result in selection bias and limit the generalizability of findings. Individuals who do not belong to faith-based and community organizations had minimal chance of participating. Religiosity is not a fixed phenomenon and is liable to change, particularly during difficult times. We have no information on changes in participants’ religiosity during the pandemic (increase, decrease, or no change) and how this might impact the study outcome. Additionally, the outcome variable was based on self-reporting of mental health issues using unvalidated questions. Though our questions were pre-tested in a fraction of the target population, and we identified no problems, there is a possibility of underreporting mental health issues in the study population. Underreporting might occur due to stigmatization and not wanting to admit to having a mental health condition. This bias could also be due to participants having never been diagnosed with a mental health disorder by a healthcare provider. Despite the highlighted limitations, our study has some strengths. It is the first in the United States to report on the mental health of African immigrants during a pandemic.

## 5. Conclusions

African immigrants experienced an increased rate of mental health disorders and employment loss during the pandemic. Implementing community-based strategies to provide social support during the COVID-19 crisis or a disaster is essential in the African immigrant community. Social support to reduce the feeling of isolation within faith-based communities could help reduce the psychological impact of fear and anxiety induced by the disaster and prevent various mental health conditions. Key stakeholders within immigrant communities and policymakers need to identify and adopt strategies to minimize employment loss, address socioeconomic issues and reduce the burden of the mental health consequences of the pandemic. These strategies may also be helpful during periods of widespread disasters. 

## Figures and Tables

**Table 1:** Sociodemographic and health-related characteristics of the study participants, African Immigrant COVID-19 Study 2021 (*n* = 260).

Characteristics	*n* (%)	95% CI for Proportion (%)
* Age in years, mean (SD)	42.0 (11.4)	-
**Gender**		
Female	157 (60.4)	54.5–66.3
Male	85 (32.7)	27.0–38.5
**Highest educational level**		
Less than bachelor’s degree	28 (10.8)	7.03–14.6
Bachelor’s degree	76 (29.2)	23.7–34.7
Graduate degree	156 (60.0)	54.1–66.0
**Employment status**		
Employed	202 (77.7)	72.6–82.8
Unemployed	58 (22.3)	17.2–27.4
**Annual household income**		
Less than USD 50,000	72 (27.7)	22.3–33.1
USD 50,000–99,999	79 (30.4)	24.8–36.0
USD 100,000 or more	104 (40.0)	34.1–46.0
Missing	5 (1.9)	0.24–3.56
**Marital status**		
Never Married	54 (20.8)	15.9–25.7
Married	181 (69.6)	64.0–75.2
Other	25 (9.6)	6.02–13.2
**Religious Affiliation**		
Catholic	35 (13.5)	9.3–17.6
Islam	90 (34.6)	28.8–40.8
Protestant or Pentecostal	103 (39.6)	33.7–45.5
Traditional and other religion	23 (8.9)	5.4–12.5
None	6 (2.3)	0.5–4.1
**Insurance**		
Yes	226 (86.9)	82.8–91.0
No	22 (8.5)	5.11–11.9
**US region**		
Northeast	124 (47.7)	2.05–7.15
Southeast	68 (26.2)	41.6–53.8
Southwest	27 (10.4)	20.9–31.6
Midwest	22 (8.5)	6.69–14.1
West	9 (3.5)	5.11–11.9
**Length of stay in the US**		
0–5 years	58 (22.3)	17.2–27.4
6–10 years	71 (27.3)	21.9–32.7
11–20 years	61 (23.5)	18.4–28.7
>20 years	68 (26.2)	20.9–31.6
**Cigarette smoking**		
Never	244 (93.8)	90.9–96.7
Former smoker or current smoker	14 (5.4)	2.65–8.15
**Alcohol use**		
Yes	70 (26.9)	21.5–32.3
No	187 (71.9)	66.4–77.4
**Body mass index**		
Underweight	2 (0.8)	0.28–1.88
Normal	57 (21.9)	16.9–26.9
Overweight	105 (40.4)	34.3–46.4
Obese	80 (30.8)	25.2–36.4
**Physical activity**		
None	71 (27.3)	21.9–32.7
Inadequate	147 (56.5)	50.5–62.5
Adequate	36 (14.6)	10.3–18.9
**Chronic medical condition**		
Yes	82 (31.5)	25.9–37.2
No	178 (68.5)	62.9–74.2
**COVID-19 infection or hospitalization**		
Yes	30 (11.5)	7.6–15.4
No	230 (88.5)	84.6–92.4

* Continuous variable reported as mean (standard deviation). Some cell numbers do not sum up to 260 due to missing data.

**Table 2:** Determinants of Incident Mental Health Disorders, African Immigrant COVID-19 Study 2021.

Characteristics	Incident Cases of Mental Health Disorders		*p*-Values
	Yes (%)	No (%)	
* Age in years, mean (SD)	37.9 (10.3)	42.7 (11.4)	0.018
* Religiosity, median (IQR)			
Organizational	5.0 (2.0)	5.0 (2.0)	0.710
Non-organizational	5.0 (1.0)	5.0 (1.0)	0.646
Intrinsic religiosity	14.0 (3.0)	14.0 (3.0)	0.595
**Religious social support**			0.020
Yes	66.7	82.4	
No	23.1	7.2	
Not applicable	10.3	10.0	
**Gender**			0.168
Female	75.0	63.1	
Male	25.0	36.9	
**Highest educational level**			0.197
Less than bachelor’s degree	2.6	12.2	
Bachelor’s degree	30.8	29.0	
Graduate degree	66.7	58.8	
**Employment status**			0.027
Employed	64.1	80.1	
Unemployed	35.9	19.9	
**Annual household income**			0.301
Less than USD 50,000	38.5	26.4	
USD 50,000–99,999	25.6	31.9	
USD 100,000 or more	35.9	41.7	
Missing			
**Marital status**			0.021
Never Married	30.8	19.0	
Married	51.3	72.9	
Other	17.9	8.1	
**Religious Affiliation**			0.126
Catholic	25.6	11.5	
Islam	25.6	36.7	
Protestant or Pentecostal	41.1	39.9	
Traditional and other religion	7.7	9.2	
None	0.0	2.8	
**Insurance**			0.103
Yes	84.2	92.4	
No	15.8	7.6	
**US region**			0.093
Northeast	43.6	50.7	
Southeast	41.0	24.6	
Southwest	10.3	10.9	
Midwest	0.0	10.4	
West	5.1	3.3	
**Length of stay in the US**			0.300
0–5 years	25.6	21.9	
6–10 years	35.9	26.0	
11–20 years	12.8	25.6	
>20 years	25.6	26.5	
**Cigarette smoking**			0.498
Never	92.3	95.5	
Former or current smoker	7.7	5.0	
**Alcohol use**			0.036
Yes	41.0	24.8	
No	59.0	75.2	
**Body mass index**			0.224
Underweight	2.6	0.5	
Normal	26.3	22.8	
Overweight	50.0	41.7	
Obese	21.1	35.0	
**Physical activity**			0.054
None	43.6	24.9	
Inadequate	43.6	59.9	
Adequate	12.8	15.2	
**Chronic medical condition**			0.627
Yes	71.8	67.9	
No	28.2	32.1	
**COVID-19 infection or** **hospitalization**			0.415
Yes	15.4	10.9	
No	84.6	89.1	

* Continuous variable.

**Table 3:** Unadjusted and Adjusted Odds Ratios of the Predictors of Incident Mental Health Disorders, African Immigrant COVID-19 Study 2021.

Characteristics	OR, 95% CI	AOR, 95% CI
* Religiosity		
Organizational	0.96 (0.76–1.21)	1.30 (0.81–2.09)
Non-organizational	0.98 (0.73–1.31)	1.33 (0.82–2.15)
Intrinsic religiosity	0.98 (0.86–1.11)	1.08 (0.92–1.26)
* Age in years	0.96 (0.93–0.99)	0.97 (0.93–1.01)
**Religious social support**		
Yes	Ref.	Ref.
No/Not applicable	**2.41 (1.13–5.11)**	**2.65 (1.02–6.92)**
**Employment status**		
Employed	Ref.	Ref.
Unemployed	**2.25 (1.08–4.69)**	1.67 (0.80–3.98)
**Loss of employment income in the household**		
No	Ref.	Ref.
Yes	**2.65 (1.32–5.34)**	**2.59 (1.21–5.53)**
**Marital status**		
Married	Ref.	Ref.
Never married	**2.30 (1.04–5.08)**	1.10 (0.38–3.16)
Other	**3.13 (1.16–8.42)**	3.69 (0.97–14.08)
**Alcohol use**		
No	Ref.	Ref.
Yes	**2.11 (1.04–4.29)**	1.64 (0.72–3.74)

* Continuous variable; OR: unadjusted odds ratio; AOR: adjusted odds ratio; CI: confidence intervals; Ref., reference group; estimates in bold are statistically significant at *p*-value < 0.05.
